# Experiences of early parenthood in and beyond the coronavirus pandemic: a qualitative study with expectant and new mothers

**DOI:** 10.1186/s12913-025-13746-x

**Published:** 2025-12-04

**Authors:** Filiz Celik, Rachel Harrad, Rob Keasley, Paul Bennett

**Affiliations:** 1 Swansea University, Swansea, Wales UK; 2 Hywel Dda University Health Board, Carmarthen, Wales UK

**Keywords:** Perinatal mental health, COVID-19 pandemic, Social isolation, Lockdown measures, Maternal and paternal mental health

## Abstract

The term ‘Perinatal’ incorporates pregnancy, birth and the postnatal period. The perinatal period is of significance in both the individual and familial life cycle, an experience shared by the wider social network and community. Yet, it can be a time where the impact of pre-existing and emerging mental health problems could necessitate specialist Perinatal Mental Health Services (PMHS) support via the National Health Service (NHS). The COVID-19 pandemic is considered to have added adverse impacts on Perinatal Mental Health (PMH), with questions on the lived experience remaining largely unanswered. The current study focussed on the experiences of a unique user group, women who were currently in receipt of perinatal mental health support during the pandemic and considered the context in which this service was received via the NHS within Wales. Semi-structured interviews with 21 expectant and new mothers were analysed using Reflexive Thematic Analysis (RTA) establishing four main themes of: 1. Disrupted Perinatal Medical Care during the COVID-19 pandemic; 2. Becoming a Parent in Social Isolation; 3. Managing Perinatal Health Through PMHS; 4. Parenthood beyond the COVID-19 pandemic. Findings indicated that the COVID-19 pandemic caused concerns and worries amongst pregnant, birthing, and post-partum women and that diminished opportunities for social support during that time created an adverse mental health impact upon them. Overall, COVID-19 appears to have a ripple effect on women and their families experiencing the perinatal period during pandemic. Participants largely attributed the causes of their poorer experiences to the pandemic by identifying it as a contributor to their heightened perinatal mental distress and at times as sole cause for their referral to Perinatal Mental Health Services. The research generates information and knowledge that highlights the unique needs of the perinatal population in future pandemics and crises, with both short and long-term implications for parents, babies and families.

**Supplementary information:**

The online version contains supplementary material available at 10.1186/s12913-025-13746-x.

## Introduction

Perinatal distress is an umbrella term referring to parental experiences of anxiety and depressive symptoms, as well as other mental health difficulties that bring a maladaptive psychological response [1]. It involves a period of time from conception to the time the infant reaches 12 months of age [2]. Distress during this period is associated with increased risk of parental mortality [3, 4], decreased quality of life, and poorer physical and mental health outcomes for the mother [5, 6], as well as long-term negative health outcomes for the child, ranging from premature delivery to increased risk of behavioural and emotional problems in later years [7, 8]. Maternal mental ill health outcomes (such as post-partum anxiety or depression) may result from hormonal changes as well as the strain of childcare and other related stressors within the individuals’ lives, from economic strains to poor social support [9, 10]. These may be exacerbated by more general factors impacting on the wider population, the COVID-19 pandemic being one such example, and whilst there is an established literature addressing the mental health impact of COVID-19 on the perinatal population (e.g. [11, 12, 13]) aspects of lived experiences, including women’s description of the impact of COVID-19 both on themselves and their families remain unexplored within this population group to the best of our knowledge.

During the COVID-19 pandemic pregnant women were advised to protect themselves against the virus, in the way that they would for any other viral infections during pregnancy and post-partum periods. Unusually however, this was combined with formal ‘lockdowns’ leading to limited access to resources normally available to such women, including antenatal classes and, perhaps more critically, immediate social and family support; the latter being primary markers of resilience and protective against a range of mental health problems, including postpartum depression [14]. This limited access to social support as well as concerns about the potential impact of transmission of the virus to unborn or newborn children were found to contribute to relatively high levels of anxiety among new mothers by Almeida et al. [15].

Little evidence of sustained institutional learning from COVID-19 [16] highlight how health systems are vulnerable to future episodes. This is particularly concerning for the perinatal population as COVID-19 exposed the vulnerability of the perinatal population via the significant impact on maternal mental health, parent-infant bonding and familial mental well-being [17]. This also calls for attention within the Welsh policy framework to prioritize perinatal population’s heightened risk during major health crisis like pandemics. The present study aimed to add to these data, providing a deeper knowledge of lived experience, by focusing on a group of women in the perinatal period who were already experiencing mental health problems prior to the time of lockdown. Specifically, this research aimed to explore the experiences of pregnant women and their families’ during this time; to provide insight and understanding into a population that needed regular and frequent support from medical professionals and hospitals; to explore these experiences in semi-rural and rural settings of Wales, and finally to generate knowledge that could aid in determining how best to provide support in the case of future pandemics or similar catastrophes.

## Methods

Qualitative methodologies enable participants to express their views, perspectives, thoughts and feelings on their own terms and in their own way, compared to more structured and prescriptive quantitative methods and was adopted to explore this novel research area.

### Ethical considerations

Ethical approval was granted through an Integrated Research Application System (IRAS) application (ID: 286205) to Wales NHS Research Ethics Committee (REC 20/WA/0243); received on the 24^th^ of November 2020. Research was conducted in accordance with the Declaration of Helsinki.

### Welsh context

This research was located in the geographical area served by two Welsh health care providers (Hywel Dda University Health Board - HDUHB and Swansea Bay University Health Board -SBUHB) during 2020–2021. During this time, the Welsh Government implemented lockdown measures, including limiting non-essential travel, preventing and limiting meeting of people from outside of one’s own household, and changes to the service delivery of the NHS. On the 17^th^ of March 2020, the NHS announced cancellation of non-urgent operations and on the 23^rd^ of March 2020 wide-ranging restrictions were imposed to protect the public and enable the NHS to respond to the needs of individuals effected by the virus. An initial reduction of restrictions was observed during the summer, followed by increased restrictions and localised lockdowns through September and October 2020, and further increases during December 2020; reductions took place during the spring and summer of 2021 [18]. Pandemic restrictions changed delivery of maternity services, in Wales these changes included hybrid provision of health care with move to digital delivery and essential care-prioritization which influenced women’s perinatal experiences [18, 19].

### Participants

Expectant and new mothers who were referred to, and in receipt of support from, Perinatal Mental Health Services through the NHS in West and Southwest Wales (specifically HDUHB and SBUHB) and their partners were invited to participate in this research.

A convenience sampling strategy was followed by sending letters to the service users of Perinatal Mental Health Service users in both health boards inviting them and their partners to take part in the study. Service users of the perinatal mental health services of HDUHB and SBUHB, who were aged over 18 years of age and had been in receipt of support of the service from at least January 2020 were sent letters of invitation to their home address from late December 2020. SBUHB sent another round of letters to service users on the 8th of August 2021. Members of the PMH services also advised their service users of the study. Content of the letters were also created as leaflets and given to service users in hard copies and digital formats. Service users either contacted the research team or passed their details to Perinatal Mental Health Teams to be contacted by the lead researcher.

Twenty-one women responded and agreed to be interviewed. They were reminded that the research was open to partners, and their partners were invited via these women. All women were cisgender and in heterosexual relationships. Two fathers came forward to be interviewed. Other fathers either did not respond or sent messages via the study participants that they did not feel their mental health problems warranted attention. Too few fathers were available to interview to permit full analysis, and these interviews were excluded from analysis.

Of the 21 participants, 20 were new mothers, only one was currently pregnant and due to give birth in the coming days. Due to the sample being predominantly post-partum, it was not possible to analyse potential differing perspectives between pregnant and post-partum women.

Interviews were conducted by lead researcher FC, a qualitative researcher and also a Highly Specialist Systemic and Family Psychotherapist. Participants were not previously known to FC but were all current service users of the perinatal mental health service.

Interviews were transcribed and analysed. (See Appendix 1: Table 1: Participant Demographics).

### Procedure

A semi–structured interview schedule (included in supplementary materials) was developed by the research team. The lead researcher brought prior professional experience within perinatal mental health services and held established connections with staff across both Swansea Bay and Hywel Dda University Health Boards. This insider perspective informed the initial stages of the research, which were further supported by a scoping review of emerging literature. In addition, the researchers drew on contemporaneous media reporting during the unfolding COVID-19 pandemic, observed public discourse on platforms such as Twitter and Mumsnet, and engaged in informal conversations with NHS colleagues regarding patient concerns. These multiple sources of insight helped shape the study’s contextual understanding and emergent focus.

Semi-structured interviews were conducted, using videoconferencing software Zoom, between April and August 2021. Participants were asked about their perinatal mental health experiences during COVID-19. (The interview schedule is available in Supplementary Materials). The semi-structured interview addressed their experiences during pregnancy and the perinatal period, their views on how these differed from their expectations and how their mental health was affected by them. Interviews were audio recorded with live close captions and lasted between 28 and 65 minutes. Transcripts were corrected by listening to audio recordings and manually correcting errors.

### Data analysis

Data was analysed using Reflexive Thematic Analysis (RTA [20]). After the transcripts were corrected and anonymised, the data was read and re-read with text units marked and broad sets of themes noted by the lead researcher (FC). Data was coded and codes were clustered together as they formed into distinct categories based on the topic/theme they generated, which then generated sub-themes and themes using a saliency analysis approach [21]. The lead researcher (female, cisgender, nulliparous, BAME, a systemic psychotherapist and researcher) completed the process of coding and generating themes, at intervals sharing the process and discussing with a key colleague (PB: male, cisgender, white British, clinical psychologist, and researcher) to ensure the data supported each category of the themes, and coded and clustered consistently

Data Analysis produced four main themes, and eleven subthemes as illustrated in Table 1.

**Table 1:** Themes and sub-themes

Theme	Sub-themes
1 Disrupted Perinatal Medical Care during the COVID-19 Pandemic	i. Exclusion of partners and emotional consequences ii. Emotional distress of being alone at the hospital iii. Home births as resistance to hospital exclusion
2. Becoming a Parent in Social Isolation	i. Unwitnessed pregnancy and the loss of rituals ii. Fragmented social support and perinatal vulnerability
3. Managing Perinatal Health Through PMHS	i. Embodied vulnerability and anxiety due to COVID-19 ii. Mental health impact of COVID-19 iii. Remote access to PMHS during COVID-19
4. Parenthood beyond the COVID-19 Pandemic	i. Bonding, isolation and maternal strain ii. Different challenges for first-time and experienced mothers iii. Fathers, bonding and mental health in pandemic

## Findings

### Disrupted perinatal medical care during the COVID-19 pandemic

Participants going through all phases of the perinatal period reported they had experienced significant adverse impacts as a result of the measures implemented to prevent the spread of COVID-19. Sub-themes described the impact of; i- **exclusion of partners and emotional consequences, ii- emotional distress of being alone at the hospital, iii- home births as resistance to hospital exclusion**

i. **Exclusion of Partners and Emotional Consequences**

To contain the spread of the virus there were limitations on who could enter health care centres. This inadvertently created a situation whereby pregnant women had to attend their antenatal appointments alone and often under uncomfortable and distressing circumstances:

*PSB01: … you have to - you have to - wear a mask and then, when you did so the nurses all wear like visors and masks and everything on but you were still like sat there with like really advice to wear a mask … the waiting room was hot, it was horrible, all the chairs taped up and you’re all just there on your own kind of like compared to the normal scans I had like in January [referring to pre lockdown scan in January 2020] it like it was it was really different, it was, trying to explain it like really lonely, you know.*

The participant’s account shows the measures aimed at preventing the spread of the COVID-19 resulted in women not being allowed to bring a support partner to antenatal appointments which then shifted these from being shared joyous occasions to moments of emotional isolation. The absence of partners heightened women’s feelings of loneliness and vulnerability as these appointments became less personal and more procedural. Intensification of pandemic related measures attributed to heightened maternal distress during COVID-19 [22, 23].

**PHD03**: *I was about nine weeks pregnant and I’m there to find out whether or not there’s a viable pregnancy. And that’s the sort of news you don’t want to be alone, for you know if you do find out its bad news, you really want to have your husband with you … or even just a friend.* *And you had to go in alone for that, completely alone, which was extremely stressful you know. I was so anxious, I was so …* *Just upset anyway at the - at the - the thought that that could be the outcome and yeah you have to be dropped off at the hospital entrance and that’s it - there’s nobody can come any further than that.*

Those with healthy pregnancies talked about their concerns for future distress, as they could not foresee what the regulations would be and how this would impact their future ante-natal appointments and birth plans. Research into trauma informed maternity care reveals the significance of having a support person, a partner, friend, relation or a doula helping reduce anxiety and support emotional resistance for pregnant women [24] whilst being denied a support partner is associated with heightened distress [25].

Participants referred to limitations on who could be at the wards, inadvertently resulting in their partners and family not being there to support them and added to this often less than optimal care from staff operating under reduced staffing due to the pandemic, Healthcare systems were under a lot of pressure and overstretched, struggling to continue with the regular care they provided, unfortunately in women’s experience this translated to feeling uncared for and even dehumanised.

[26, 27]

ii. **Emotional Distress of being Alone at the Hospital**

All participants described the adversity of their experiences of going to hospitals, from outpatient departments to admissions, for birth and post-partum care. Unfortunately, reduced quality of medical care could lead to emotional experiences of high levels of distress and even psychological trauma.

**PHD03:** And he[her husband] had to leave, and that was it, you were - you just had surgery you lost a lot of blood and you’re there with a new born baby take care of, you know. I mean this is the situation, and you are responsible for yourself**. …** I know, technically, the midwives, are there and the staff are there, but they are so busy, and so rushed off their feet that you can’t really get hold of them every time you need something. I mean you do need a lot at this point. **…** your baby is absolutely screaming their heads off this tiny little new-born and they’re right next to you and you can’t really even twist your body to get them it’s a horrible situation to be in really, really, you know horrible and you feel very, very helpless.

This account details the embodied vulnerability of postnatal care from lying alone, to being in physical shock and being unable to care for her newborn. Overall, the toll of being denied her husband’s support during a traumatic birth led to a profound sense of abandonment and helplessness. During COVID-19, as the demand for medical care increased, availability of health professionals decreased due to staff contracting COVID-19 or requiring shielding following direct exposure to the virus. For many women, whilst staff were present, their limited availability meant that they had to manage post-surgery recovery and care of a newborn without timely support. The impact of this was tangible for women as in the absence of this support, they were scarred with memories of helplessness.

Recent research reveals that strained maternity services during pandemic compromised safe and person-centred service delivery [26, 28] and lack of in-person support for pregnant women is linked to perceived neglect and increased maternal distress leading to presentations of the symptoms of post-traumatic stress [29]. This is also alarming as women may internalise circumstances of this gap in service delivery as a sense of failure and even shame [30].

iii. **Home births as Resistance to Hospital Exclusion**

One of the challenges faced by the participants was giving birth under lockdown rules. Owing to a fear of giving birth in the absence of family support, six participants reported considering changing their birth plans to home births so that their partners could be with them throughout the process. Due to complications such as premature births or caesarean, only two women could go ahead with a home birth. Community births are sometimes considered riskier than hospital births [31], yet COVID-19 sparked an interest in giving birth at home [32].

**PHD10:** I wanted to have a home birth, because I hadn’t had a positive experience last time, but - but - my decision was influenced by worries about whether my husband, will be able to be in hospital with me and worries about what we would do with our other child while I was in hospital.

Anticipation of the experience of birth is common amongst pregnant women and can affect their emotional wellbeing in the post-partum period [33]. However, as uncertainty about how pandemic related restrictions would affect their birth experience heightened, concerns for risk shifted beyond the biomedical aspects of the birth into uncertainty about the psychological aspects of the experience, deepening emotional and relational vulnerabilities with tangible feelings of anxiety and other emotional distresses for the participants.

**PSB03:** But anyway, definitely that was that was the one non-negotiable was, I wanted him to be with me the whole time when we knew that., he was going to be separated from me, straight after we change to having home birth so we booked and set it up with a great time doing that we figured out what was going to happen we figured out the timings from our house hospital, checking the statistics and everything.

### Becoming a parent in social isolation

Participants talked about the difficulties of becoming a parent in social isolation during various lockdowns. Two sub-themes emerged in this context: i- unwitnessed pregnancy and the loss of rituals, ii- fragmented social support and perinatal vulnerability

i. **Unwitnessed Pregnancy and the Loss of Rituals**

Pregnancy, birth, and post-partum periods are phases of life that are celebrated with rituals such as maternity photoshoots, baby showers, antenatal classes and introducing the new baby to the world. These rituals not only celebrate the birth of a baby and parenthood but support psychosocial adjustment to motherhood. The COVID-19 pandemic inhibited the practice of these well-established rituals, replacing collective joy with isolation and anxiety as risk of transmission to pregnant and post-partum women posed a great risk and required them to shield [34, 35]. Participants expressed how lockdown measures caused a social and emotional deprivation for women:

*PSB15: Especially because everyone’s anxiety was through the roof anyway, while being pregnant, because you couldn’t you couldn’t go outdoors I see we’re locked down because the point where we need to go for five minutes Ollivier, walk every day, and that was that.*

This participant expresses the toll of lockdown and heightened distress via describing a simple activity such as going outdoors, a source of regulation and release becoming inaccessible.

*PHD01: there was times I was really looking forward to my antenatal appointment because it was literally, the only socialization and the only trip out of the house, and then the midwife would call and say oh we’re gonna have to do it over the phone … . There was no hypno birthing classes, which I was hoping to attend, there was no antenatal classes, which I would have attended … There was no pregnancy yoga which I plan to attend basically everything was cancelled, and it was it was quite a solitary experience; to be honest and the midwife was always on the end of the phone, but you know it’s not the same …*

Women talked in detail about how lockdown limited their lives, not only their daily activities but overwhelming feelings of solitude and disorientation. Rituals around pregnancy, birth and post-partum are acknowledged as protective factors for women, reinforcing their new identity as parents and helping normalise the physiological and psychological changes they underwent [36, 37]. The loss of rituals and taken for granted interactions removed the symbolic and relational structures that supported their transition to motherhood impeded on their psychological preparation to welcoming a baby and parenthood. The restrictions of lockdown were beyond being denied daily activities but extended to being denied safe opportunities for the creation of lifelong memories. Long term effects of these continued to be observed within the perinatal population [12].

ii. **Fragmented Social Support and Perinatal Vulnerability**

Existing literature on the post-partum experiences of women indicates that those who have access to social support from family and friends fare better with depression in the post-partum period [38, 39]. Primiparous women expressed that they often struggled to make sense of their experiences; from judging if they were progressing well with their pregnancy to managing their expectations of birth and post-partum periods.

**PSB15**: I didn’t know as being like it my first my first pregnancy, I wasn’t sure what was normal and what - what - wasn’t obviously I understand because of COVID19, nothing was normal you know everything had been adjusted anyway, and I understand that, but. I also I wasn’t sure when exactly I was supposed to be having check-ups and seeing the midwife and having scans and it was actually my family like or Facetime over message and that were like have you seen your midwife, and I was like no not seen her. I think it was 10 weeks, I went without seeing her and they were like no, you need, you need to report, you need to ask what what’s going on and to be fair, without them saying I probably wouldn’t have done anything.

Primiparous women reported difficulty interpreting their own experiences in the absence of guidance and reassurance, lack of in-person care leading to feelings of invisibility and confusion. On the other hand, multiparous women had the advantage of knowing about the process owing to their previous experiences. Comments such as “because this is my second child, I already knew how to …” were amongst repeated comments. Familiarity with the rhythm of pregnancy and post-partum state of self seemed to help buffer against some of the challenges. However, multiparous women commented on the negative impact of a lack of social interaction and the challenges of managing a newborn whilst taking care of an older child who could not be sent to nursery, or of the challenges of managing the home schooling of older children. Although people used online platforms to connect, linking in digitally did not fully mitigate the complete social isolation.

*PSB10: Obviously, having one child, you know, I know I know where it’s like I know the baby blues say know, everybody gets it and that’s normal, but I knew this time it wasn’t normal and I think a lot of it was triggered by obviously lockdown and you know, we had COVID and we were struggling …*

Here, previous perinatal experience becomes a resource, highlighting the toll of isolation and awareness onto their exacerbated symptoms owing to COVID-19 and requiring them to access more social support that had been dwindled owing to COVID-19.

*PHD03: I think mentally long-term mental health conditions are definitely going to arise as a result of it, you know unless you’re super woman who is, you know, the most mentally stable person who would never be affected by anything frankly you’ve got to be a little bit on the psychopathic scale …*

These accounts reinforce the critical importance of social embeddedness in the perinatal period, reinforcing the protective role of support networks [38] and how the erosion of such networks, whether clinical or communal, can worsen the psychological burden. The lack of opportunities to be seen, heard, guided, or celebrated during this life-altering transition resulted in a psychosocial void for many participants, posing potential risk for bonding, and long-term mental health.

### Managing perinatal health through PMHS

Participants reported the challenges posed by COVID-19 to their mental health and to their receiving support from PMHS. Three sub-themes informed this theme: i- i- Embodied vulnerability and anxiety due to COVID-19, ii- mental health impact of COVID-19, iii- Remote Access to PMHS During COVID-19

i. **Embodied vulnerability and anxiety due to COVID-19**

The prevalence of anxiety disorders is relatively high among the maternal perinatal population [40]. Accounts of the participants reflected on how their anxieties emerged or were exacerbated by the COVID 19 pandemic. Women referred to their need to shield from COVID-19 and how in doing so, their mental health suffered because of a lack of everyday interaction and social support.

**PHD01**: And I didn’t even go to the shops, because I was worried about being pregnant and it was so early that there wasn’t a lot of research, and pregnant women were being told to be more cautious.

Scientific uncertainty about the potential impact of COVID-19 on pregnant women lead some women to interpret the risk as acute and imminent and as this participant explained caused behavioural restrictions beyond government advice.

**PSB04**: Well, I, I feel like I can’t take her anywhere, you know we don’t take her into shops. You know just the everyday stuff. So that’s really you are really restricted and I - I’m really, I get quite anxious about it, I mean it’s just family, but I get anxious about family members come into the house because.

Participant accounts echo and reinforce the existing empirical evidence that the risk of contracting COVID-19 exacerbated their anxiety for their pregnancy and babies, to the extent that they negotiated risk by narrowing their social and physical worlds. As Vanstone et al. [41], suggested that biomedical framings of vulnerability during the pandemic translated into self-regulatory practices that exacerbated anxiety and curtailed opportunities for social support and accumulated into psychological dysregulation. As such the uncertainty during the pandemic heightened personal responsibility, which has significant implications for maternal mental health.

ii. **Mental health impact of COVID-19**

Whilst the pandemic was found to have varying levels of adverse impact on the mental wellbeing of the general population [17], pregnant and post-partum women were identified as at heightened risk of negative impact by the restrictions imposed to prevent disease spread [34, 35]. Accounts of the research participants highlighted the inescapability of their mental health suffering.

*PHD03: I think mentally long term mental health conditions are definitely going to arise as a result of it, you know unless you’re super woman who is you know the most mentally stable person who would never be affected by anything frankly you’ve got to be a little bit on the psychopathic scale so something like that, not to affect you mentally. I’ve got to be in that position and given us like that and to have absolutely no support and have to hold it together and walk out of that hospital and find your car or find your partner who’s waiting outside.*

*PSB02: … then I started like suffering a little bit because I was seeing my partner less and less because of the outbreak in his workplace, he was having to cover like loads of shifts so I was on my own even more … and I will say that the maternity care during COVID has been pretty awful and … you know I worry about what - what’s that done to their mental health, mothers who didn’t have their partners with them for the scans? …*

iii. **Remote Access to PMHS During COVID-19**

PMHS changed its service delivery to hybrid and introduced videoconferencing to promote access to services during COVID-19. Participants’ accounts overwhelmingly demonstrated that they found the support of PMHS beneficial. Some participants commented on PMHS being an underused service and attributed this in part to hesitancy of women asking for mental health support for what is regarded as a celebratory phase in their lives and in part to the lack of knowledge about a comparatively new service. PMHS as a sub-speciality psychological service is still a very young service in the UK [42] and a national clinical PMHS Lead for Wales was only appointed as recently as 2019.

*PSB12: Just that I think it’s the perinatal mental health services, a very undervalued, I suppose, really, especially in times like COVID and I think that they’ve done a wonderful job of being there for expectant mothers throughout and they’ve given a lot of support and I think that more women shouldn’t, they shouldn’t be frightened to ask for how - they shouldn’t be frightened to - you know, admit that there’s something wrong with feeling low and share so anything because services are there to - not to judge and, yes, I just like to add that in that’s okay.*

The account of the participant illustrates how women themselves can become advocates for PMHS, challenging stigma and encouraging engagement, a role that has been emphasised in the literature calling for greater normalisation of mental health support in maternity contexts [43]. PMHS adapted their services in line with the COVID-19 regulations and began routinely offering remote provision of services via telephone and online platforms. Accounts of the participants reflect that transformation of the services to online was well received by some of the participants as it helped their anxiety about going out to meet people, as well as new anxieties about exposure to COVID.

*PHD07: Fantastic, absolutely fantastic and, obviously, it was initially it over the phone when I spoke to … Initially it was just a telephone interview, how I was feeling and where I was, but I found that that was easier because I hadn’t had to go anywhere and meet a stranger and talk to a stranger face to face and so yeah that was brilliant and then we had a program run by a lovely lady called [name omitted] and that would give us just basically emotional coping skills and different strategies that we could - we could do is at all I’m not just myself, but you know as a family to - to - help manage*

Accounts of this participant highlighted the remote services enabling better access to PMHS and removing the difficulty of travelling to the clinics, and for some removing the anxiety of meeting a new person. This reflects how digital access can ease the distress of accessing services for those with anxiety and mobility challenges [23].

However, not all participants referred to benefiting from digital and other forms of remote access and commented on their feelings of being disadvantaged by the remote service delivery, and made comments that they would find it easier to engage if the services were offered face-to-face:

*PHD02: It was a big thing, because I think if I’d have been able to go in, it would have been easier to see that there was problems and those things I needed to talk about so I didn’t take the advantage of that service that I should have because it was remote, that was the trouble.*

Comments from participants highlighted how remote services may become a barrier to engagement and perhaps contribute to under-utilisation. This reflects critiques of digital healthcare that caution against assuming equivalence between remote and face-to-face methods, as some women may feel more supported in the immediacy and embodied presence of in-person interactions [44].

### Parenthood beyond the COVID − 19 pandemic

An Attachment Theory informed approach suggests that heightened distress due to the COVID-19 pandemic might have long-term impact on parent-child relationships [45]. The attachment relationship between parent and offspring starts developing from pregnancy [46] and can be impacted by major disasters due to their frightening and life-threatening nature [47]. Research already suggests an adverse impact of COVID-19 on family systems through the added distress of COVID-19 and social isolation on mothers during the perinatal period [36, 48]. Participants in this study talked about the specifics, about their concerns around parenting during the COVID-19 pandemic and raising their babies in isolation. Three subthemes formed this theme: i- Bonding, Isolation and Maternal Strain, ii- Different Challenges for First-time and Experienced Mothers, iii- Fathers, Bonding and Mental Health in Pandemic.

i. **Bonding, Isolation and Maternal Strain**

Twenty out of 21 participants were women who had already given birth to their babies during the COVID-19 pandemic. A central theme emerged around difficulty of caring for newborns in profound social isolation created by the COVID-19.

Participants expressed concern that they were bringing their babies up in the absence of communal social experiences being denied going to mother and baby events, limited celebrations of the arrival of the babies, missing the involvement of the extended family or friends. Mothers referred to their “clingy” babies and expressed concerns that the lack of interaction with others could have potential developmental impacts on their babies’ development. One participant, for instance, explained:

*PHD01: it’s hard to say, and I think I don’t think we’re going to know for a few years what the impacts of this will have been on the babies themselves, and I think like for me and my baby, we’ve got a very strong bond and I think, maybe there’s an element he’s at an age now where he recognizes strangers he’s got a strong attachment to me, so I think a lot of it is normal, but I think there’s also an element of when there’s a lot of people or people he doesn’t recognize he does get a bit sensitive about it, and I think part of that is because he hasn’t ever kind of had his - you know - he’s never been to a park he’s never been to a baby group he’s never done … . Say Oh, can you take a minute for me to go to toilet, so you know so from a practical point of view, it had an impact because I felt like I was doing absolutely everything by myself and from a mental and emotional point of view, it was – it- it’s been really hard because I’ve really wanted to share him. And I wanted to spend time with friends and family, and it’s just not been possible.*

This narrative reflects a complex interplay between maternal bonding, social isolation, and emotional strain. Potential negative impact of social isolation during the perinatal period in increased maternal distress, delayed infant socialisation, and reduced access to informal support systems [22, 49]. Lack of attachment diversity limits the opportunity for infants to develop secure relationships with multiple caregivers beyond the mother [50]. It is also possible that lack of attachment diversity intensifies the burden on gendered expectations of motherhood, to satisfy the baby’s need and intensifies mothers to internalise more pressures on themselves in caring for their babies.

ii. **Different Challenges for First-time and Experienced Mothers**

Accounts of the participants indicated that when relational, social and institutional support for pregnant and post-partum women disrupted by lockdown measures, this posed different challenges of COVID-19 for primiparous and multiparous women.

Primiparous mothers reported their struggle with a lack of support adding to their lack of confidence about parenthood during their novel experiences of pregnancy, birth and managing a new-born. Without prior reference points, many reported struggling to interpret what was “normal,” and the loss of face-to-face reassurance from midwives and social networks undermined their confidence in pregnancy and early parenthood.

**PSB15:** *“Yeah, and I didn’t know as being like it my first my first pregnancy, I wasn’t sure what was normal and what what wasn’t obviously I understand because of covid 19, nothing was normal you know everything had been adjusted anyway, and I understand that, but. I also I wasn’t sure when exactly I was supposed to be having check-ups and seeing the midwife and having scams and it was actually my family like or facetime over message and that were like have you seen your midwife, and I was like no not seen her. I think it was 10 weeks, I went without seeing her and they were like no, you need you need to report, you need to ask what what’s going on and to be fair, without them, saying I probably wouldn’t have done anything.”*

This testimony highlights the heightened vulnerability of first-time mothers, who lacked both experiential knowledge and professional guidance, relying on experiences of family members through digital platforms. Such accounts indicate that first-time mothers may have been more adversely impacted by the pandemic restrictions owing to their need for reassurance and support to navigate this new journey [51, 52].

In contrast, multiparous women often described drawing confidence from prior experiences of pregnancy and birth, which served as protective resources against uncertainty.

*PHD10:* “I think if it had been my first pregnancy, I would have been quite anxious but, with it being the second, I guess I sort of knew a bit more and it was less, slightly less worried, for that reason.”

Here, prior experience appeared to reduce anxiety, providing multiparous women with a comparative framework to interpret symptoms and navigate service changes. While multiparous mothers drew confidence from previous pregnancies, they had additional stresses of caring for older children under lockdown conditions and the overwhelming pressures of balancing multiple roles within the household, including childcare, home-schooling, and domestic responsibilities alongside caring for a newborn.

*PHD02: We found ourselves in this position, your childcare problems or your child care problems and I said, well, I can’t - I can’t - leave him unattended, you know, have to - have to be there … incredibly difficult and there was lots of arguments within the home, we were in with each other’s way all of the time, and I was really, really tired with the pregnancy, because I’m a little bit older and - and - it was just really difficult I couldn’t give the children, what they needed I couldn’t do the home schooling that they needed me to do on the level that … . … . and like when I first - when I was pregnant a few other mums I said Oh, you know the kids can come around after school a couple of days a week, and have dinner, you know so you’ve got longer and things like that, on the weekend, they can come around, but obviously none of that could happen.*

This narrative illustrates how, owing to longer duration of the maternity leave, COVID-19 amplified gendered expectations of care, placing disproportionate pressure on mothers to manage domestic, educational, and emotional labour, simultaneously [53]. The loss of informal support networks, such as reciprocal arrangements with friends and neighbours, further intensified this burden, leaving mothers feeling isolated and overstretched. Narratives of the participants reveal that COVID-19 accentuated different vulnerabilities for primiparous and multiparous women. Despite differences in experiences, both primiparous and multiparous perinatal women faced heightened mental health risks during the pandemic, shaped not only by biomedical vulnerability but also by relational, cultural, and structural conditions [17, 54].

iii. **Fathers, Bonding and Mental Health in Pandemic**

This research was originally designed to involve both parents but was unable to directly analyse fathers’ experiences due to small response rate. However, during interviews most mothers talked about a negative mental health impact on their partners, particularly the experience of loss and grief experienced by partners because they could not be present at events including scans, early parts of labour or missed out on spending time with their partners and newborn in the hospital.

*PSB08: We suffered as a family because things that - that - got taken away from us, so my partner is only properly started bonding with my daughter now. You know the dad, you know when she was born okay go now you’re allowed two hours, two hours he could have been there for, but he was there for about an hour and he had to go home and - and - then like we’ve always said, like we both made her, I did not just make that baby, this is his daughter as well, you know, and he’s always said, you know how it’s and it has affected him, you know we asked, and he you know he hasn’t been that - that - that he you know he is a brilliant now, but you know, he was too scared to do anything, you know if it was the same you know whether it’s just our first daughter I don’t know you know.*

This quote captures both immediate and long-term impact of paternal exclusion. The sense of shared parenthood expressed through “we both made her” was challenged as the COVID-19 related restrictions placing mothers in the role of “primary parent”. Multiparous women, in particular, were able to compare their current experiences to previous pregnancies, emphasising how exclusion during the pandemic starkly contrasted with earlier, more inclusive antenatal and birth experiences.

This participant demonstrates how delays in bonding of the dads and babies could diminish paternal self-efficacy, evidencing relational consequences.

*PSB06: Because he’s already struggling with mental health, he felt like it was more difficult to connect with the baby, it was very, very protective over us but compared to my first pregnancy, with my eldest he was able to go to every appointment, he was very involved and now with this one, because of the pandemic. He couldn’t go to any of the appointments and all he could really get what’s the updates, I will bring him when I come home and share it with pictures.*

As the structure of daily life had shifted to fathers working from home or being on furlough, this emerged as a benefit to families, in contrast to ‘non-pandemic’ times. Compared to two weeks paternity leave, a new father could spend more time with the newborn, bond better with the child, and better support their partner.

*PSB03: I think it’s been good, and because he’s been able to be home and he said how grateful, he is that he’s could be around to experience now um but I know he felt utterly useless when I was in hospital, I mean there was he couldn’t even do the being there for me, because he had to be at home.*

This statement reveals the ambivalence of fathers’ experiences. Whilst exclusion from hospital-based care left fathers feeling powerless, unable to “be there” for their partners at critical moments, furlough and remote work facilitated involvement at home, allowing fathers to spend more time with their babies and to support their partners in ways not typically possible under conventional leave policies. There is a growing research interest and evidence base suggesting that longer paternal parental leave has a positive impact on both paternal [55] and parental mental health [56]. These self-reports of mothers add valued perspective to the debate about changes to parental leave which would enable both parents to benefit from time to adapt to parenthood and bond with their babies, demonstrating how increased paternal presence at home can strengthen bonds and provide practical and emotional support.

## Discussion

Participants of this research, who were all patients of the PMHS of the NHS during COVID-19, talked at length about their experiences of being pregnant, giving birth and new parenthood during the pandemic. These descriptions of their lived experiences were transcribed and analysed, forming four main themes with sub-themes. Participants described how COVID-19 heightened their distress during the perinatal period due to the fear that they were unable to protect themselves and their babies from contracting the virus and adjusting to life with such fear at a time when their sources of resilience, including practical and social support from their families, friends and communities, were significantly reduced. In this study, deprivation of such support due to COVID-19 prevention measures was identified as a contributor to feelings of loneliness, low mood, and anxiety during the perinatal period.

### Learning from the COVID-19 pandemic

COVID-19 is no longer a pandemic, and the alert levels are suspended [18], the process of understanding the impact of the COVID-19 remains underway. Learning from this will help us understand the future experiences of the current population and even experiences of future generations. This is imperative as the lessons learned from smaller scale illness outbreaks, including Severe Acute Respiratory Syndrome Coronavirus Epidemic (SAR-COV-1) [57], Middle East Respiratory Syndrome Coronovirus (MERS-CoV) [58], and Ebola Virus Disease (EVD) [59] seemingly did not effectively inform practices of pandemic preparedness for COVID-19.

Rapid fading of the collective memory of the pandemic [60] and what Bourrier and Deml [16] called ‘pandemic fatigue’ is considered very alarming given how the world maybe very close to experiencing a new pandemic at any time whilst few lessons have been learned. Collective forgetting of the COVID-19 pandemic is criticised for allowing awareness and investment to fade and political will to diminish as the immediate crisis subsides [61]. This erasure of memory has significant implications for perinatal mental health, as COVID–19 demonstrated the heavy burden of the pandemic on women’s and maternal health. Whilst the long-term impact of the COVID-19 will be continued to be identified, forgetting of the pandemic and lack of institutional and collective preparedness continues to leave populations vulnerable to future pandemics.

### Management of the pandemic and deprivation of social support

Analysis of the data indicated that the perinatal period was marked with a heightened sense of uncertainty. Descriptions of the uncertainty reveals that new mothers were deprived of the assurances which would typically be provided from their social support networks; families, antenatal support groups, and mother and baby groups, all of which became harder to access or completely unavailable during the pandemic.

Differences in the levels of need for social support for primiparous and multiparous women were evident. Primiparous women reported lower levels of self-efficacy about the process of pregnancy and impending parenting compared to multiparous women who had already experienced it. Reported levels of lower self-efficacy in women were particularly concerning as expectations of low-self efficacy in perinatal women can correlate with concurrent experiences of depressive symptoms and anxiety [62]. A lack of support was particularly problematic for primiparous women. The benefits of such support can range from contributing to a healthy pregnancy to a more positive birth and postpartum experience [63, 64].

Pregnancy, birth and having a new baby are milestone experiences and are marked with certain societal rituals. Confinement created by the pandemic prevented such rituals, with participants poignantly describing how “no-one saw them pregnant”, one of them likening their experiences to the pregnancy of women from the Victorian era, hiding their pregnant bellies. Whilst digital platforms provided some connection, participants noted that video calls fell short of replicating in-person interactions which further amplifies feelings of isolation. This absence of embodied social contact compromised them being recognised as pregnant, adding to their distress.

The adverse impact of the lack of support from the social support network adds to the findings of existing studies, suggesting an association between parental support and maternal self-efficacy and lower depressive symptomology [65], perceived social support from friends and significant others contributing to reduction of depression in post-partum women [2, 39, 66]. These findings underscore the importance of policy responses in the post-pandemic period that prioritise rebuilding parent–infant group activities, expanding access to early years services, and formally recognising the increased emotional burden on mothers during COVID-19.

### Reframing risk in birth planning and family care

Whilst primiparous women reported difficulties around navigating unknowns in the absence of usual social support, multiparous women reported further causes for anxiety and perinatal mental distress arising from the need to look after and home-school older children whilst simultaneously preparing or adjusting to the parenting of their new-born. Under non-pandemic circumstances older children would typically be in school or looked after by family and friends, which would provide respite for parents. Some participants talked of considering or indeed changing their birth plans to home births so their partners could be with them, but multiparous women also referred to challenges of arranging childcare during COVID-19 as a factor in considering or deciding on a home birth. Increased number of home births were recorded during COVID-19 [67–69]. Unfortunately, in the UK some NHS Trusts suspended their homebirth service during COVID-19 [70]. No such bans were recorded for NHS Wales trusts. Home births were recorded to increase from 2.1% in 2019 to 2.3% in 2020 and 3.2% in 2021 and this increase is attributed to COVID-19 related changes to birth plan [71] and [72].

COVID-19 challenged the expectant mother’s view of hospitals as a safe place, with home births now being reconsidered as hospitals represented a site of uncertainty and emotional fragility. Such responses can be read as a form of relational resistance, where women navigated between public health restrictions and their own need for continuity of care and companionship in birth [73]. This highlights the need for reconsidering policy frameworks that does not treat emotional and relational safety as equivalent to biomedical safety. Changes to birth plans, whether they were considered, carried out after consideration or made as a result of complications or emergencies during COVID-19 might need to be considered, as it is concluded by Liu et al. [74], unexpected changes to birth plans during COVID-19 may contribute to depressive symptoms and even PTSD among birthing mothers.

### Exclusion and barriers to paternal involvement in perinatal care

A strong narrative was presented by the women about the practical and emotional difficulties created by the exclusion of their partners from scans and other hospital appointments and admissions. The research aimed to capture the lived experiences of the fathers too and further invitations were extended to them via their spouses and partners. Unfortunately, due to only two fathers coming forward to be interviewed, there was insufficient data to analyse in equivalent depth. The literature has indicated the importance of research in this area, indicating, for example, that although fathers experience psychological distress during the perinatal period the stigma around seeking support causes self-doubt and questioning of legitimacy of such needs [75]. Wider research has observed an increase in the levels of anxiety and depression amongst paternal population during the pandemic [76, 77]. The current research highlights the difficulties fathers may feel in engaging in research: more opportunities are needed for fathers to challenge and change notions around masculinity and fatherhood [77, 78].

The low level of male participation in the current study is, in itself, a meaningful finding that calls for further inquiry into the barriers men may face when engaging with research in this domain. Future studies are needed to explore these challenges more deeply and to identify effective ways of supporting fathers during the perinatal period. This is especially important in light of growing evidence that paternal mental health difficulties can have broader implications for family well-being [79]. A further systemic issue lies in the lack of formal diagnostic recognition for paternal perinatal depression in major classification systems such as the DSM-5. This omission complicates cross-study comparisons and contributes to the broader invisibility of paternal experiences in both clinical and research contexts [80]. Although the present study could not incorporate paternal accounts into the main analysis, it is important to note that women spoke of benefits of dads being at home -either via work from home or furlough- which they believed father’s involvement in childcare positively influenced attachment between fathers and the babies. Which adds to evidence base that paternal involvement in perinatal period warrants more research attention [81].

### Attachment bonding and developmental risks during COVID-19

Attachment-related concerns were raised by the participants. Mothers described their babies as being overly clingy to them and expressed concern that the babies were deprived of meeting wider family members, grandparents, friends or other babies and interacting with them. Multiparous women compared their earlier perinatal experiences to highlight the differences between their previous and COVID-related experiences. They talked about their inability to attend events and activities with their babies, descriptions like “my baby hasn’t met her grandparents” echoed throughout, highlighting how babies born during COVID-19 had much narrower experiences of the wider world.

These accounts reflect how COVID-19 curtailed relational opportunities for babies with potential impact on their development. The ability of children to develop cognitive executive functions is directly linked to the stimulating activities that they engage in with their caregivers [82], and a reduction of the sources of support from the social environment reduced the capacity of parents during COVID-19 to provide this [83].

Further challenges emerged for the parents who experienced complicated births or had neonates who needed immediate medical attention and were admitted to NICU (Neonatal Intensive Care Unit). A NICU admission combined with the limited parental contact allowed during the pandemic added to adversities experienced by both neonates and parents and could contribute to a detrimental effect on the attachment relationships between them [84]. The impact on attachment can continue to adversely influence the later mental and physical well-being of infants [85].

The relationship between heightened perinatal distress and poorer bonding between mothers and infants, which is largely studied through a Maternal-Foetal Attachment (MFA) perspective, was also evident. Existing research underscores the crucial role of social support in preventing poorer MFA [86] and a correlation between prenatal anxiety and perceived lack of social support contributing to poorer MFA [8, 87, 88]. This poor MFA would directly impact on the infants’ wellbeing [89, 90]. Moreover, poorer attachment relationships with infants could affect the future mental being of the infants negatively and adding to child, adolescent and adult mental health difficulties [82, 91]. Participants’ reflections therefore highlight the multi-layered ways in which the pandemic shaped parent–infant relationships—not only in the immediate postpartum period but potentially across the life course of the child.

### Limitations and recommendations

This research presents a snapshot of the challenges and experiences of mothers during the COVID-19 pandemic through analysis of the narratives of women (expectant and new mothers) who were referred to and in receipt of support from Perinatal Mental Health Services through the NHS in West and Southwest Wales. The views and opinions expressed in this research are specific to the women who took part in the research. Hence, this research is limited in the extent generalisations can be made from these views and opinions, yet they are strong voices of women who came forward to make their experiences known to enable improvements in perinatal mental health care delivery which will be of benefit to the wider population.

As only two dads came forward for this research, their interviews were excluded from the analysis as it was not possible to achieve meaning saturation [92]. It is important to understand the negative impact of perinatal period on fathers’ mental health [93] to produce systemic support for both parents. Extended paternity leave is said to help improve fathers’ psychological well-being [55]. Whilst this topic requires more research, it was poignant that the mothers taking part in this research commented that fathers being at home, owing to furlough or work from home as a result of COVID-19, were beneficial as they had support with caring for the infants and fathers being more involved and bonding with their babies. The experiences of fathers require further investigation to build on emerging evidence [81].

The lack of participant diversity, specifically that all participants were cisgender women in heterosexual relationships and that no fathers participated, is a limitation to this study. However, given that PMHS accepts referrals for women and the focus of the research was experiences of women and their families, the women only demographic constitution of the study did not impact reaching thematic saturation. A sample of 21 participants is considered robust for qualitative research and allowed for the development of rich, credible, and well-saturated findings [94]. However, this demographic homogeneity may limit the transferability of the findings as perinatal mental health is increasingly understood to be shaped by intersecting gendered, cultural and structural inequalities. The absence of paternal and LGBTQ+ voices in this study calls attention to difficulties in inclusion of more diverse samples both in academic literature and service delivery [95]. Furthermore, men, gender-diverse parents and same sex couples may experience perinatal distress in distinct ways and may face difference challenges in accessing appropriate care and support [75, 96].

The absence of independent coding by multiple researchers may be regarded as a limitation, given that such practices are widely endorsed within the qualitative research literature (e.g. [97]). However, both researchers were cognisant of this limitation and adopted a reflexive stance throughout the analytic process. Ongoing dialogue was maintained regarding their own positionalities, assumptions, and affective responses to the data. In line with a constructivist epistemology, the findings are understood as a co-construction of meaning between the researchers and the data [98, 99]. Following a rigorous and dialogical process, it was collectively agreed that re-coding the data would not substantively enhance or alter the analytic outcomes.

### Conclusion

Research emerging about the impact of the pandemic indicates the highly detrimental effect of this period on maternal mental health, from heightened symptoms of anxiety [2, 100] to post-partum depression and Post-traumatic Stress Syndrome (PSSS) [101] and is likely to contribute to poorer outcomes for the infant and for familial mental health. Perceived social support is linked to better maternal mental health through decreased parental stress, increased parental responsiveness and stimulus, reduced maternal depression and anxiety [48]. Further, perceived poor maternal support is linked to negative impacts on the infant into childhood and adolescence with outcomes such as poorer cognitive abilities and increased child mental health problems [102].

COVID-19 emerges as a major challenge for women and their families who experienced pregnancy, childbirth and post-partum period. Although the participants did not link all their emotional distress and mental health problems that led to referral to PMHS directly to the pandemic. However, they did note that the circumstances and environment which they experienced their perinatal period heighted any distress they were experiencing. The increased need and demand for mental health support occurred at a time that, paradoxically, PMHS needed to limit their interventions due to the pressures the virus presented [54]. Inaccessibility of social support, leading to adverse mental health outcomes emerged as an overarching theme within this research.

This study highlights the profound impact of COVID-19 for perinatal mental health in Wales, emphasising the need to focus on policy, implementation and future research in Welsh context. Initiatives such as the Early Years Delivery Plan [103] and recently published 2025–2035 Mental Health and Wellbeing Strategy demonstrate Welsh Government’s policy initiatives to promote a person-centred approach, recognising the wider social determinants of mental health, and emphasising early childhood and infant–parent relationships. The changes in medical care created an environment that restricted the quantity and quality of medical care as well as social support for women. Both outpatients and inpatients, were negatively impacted due to virus-related staff shortages impacting both medical care and psychological support through social interactions made in medical settings, meetings with medical staff were moved to the telephone or videoconferencing meetings. These findings call for adapting service delivery in major crisis situations. Narratives of the participants reveals that shift to remote PMHS provision was met both with appreciation and hesitation. The Welsh Government acted quickly to initiate support during the pandemic via an “Attend Anywhere” system where healthcare was digitised [19]. Whilst it reduced the barriers to access and helped with continuity of care, it also limited the depth of engagement. These mixed accounts suggest this hybrid provision could especially help those living in rural areas and may have travel times in excess of an hour to reach a relevant clinic or hospital, whilst also calling attention to digital poverty in rural areas. Future pandemic preparedness needs to take into account geographical location related factors around resource access amongst perinatal populations [104]. 

A perinatal perspective calls for focused attention on the impact of the COVID-19 pandemic on mothers, infants and families, not only to ensure continued support for those who experienced the pandemic but also to draw lessons for future disaster preparedness and response [17, 54, 67]. The emerging research base around babies born during COVID-19 is highlighting a reduction in infant development score [105]. Specifically, parentally reported outcomes indicated potential deficits in the social communication skills of babies (Bryne et al. 2023) and small albeit notable differences between pre-pandemic and pandemic born infants, indicating increased risk of delayed development for pandemic babies [106]. The Welsh Government addressed these concerns through its *Flying Start* programme, (providing early years childcare support from 2 years old onwards), and enhancing health visiting, speech, language and communication support and parenting support, although the rollout of this has seen delays [103].

Management of future epidemics and pandemics need to prioritize the needs of the perinatal population as although not physically ill, they do require frequent and intense medical intervention along with social support. Providing adequate support during the perinatal period has huge benefits. Such support could help foster better attachment between babies and parents and other care givers and help prevent mental health problems not just for parents but for babies through infancy, childhood, adolescence and adulthood [107]. This research provides vital insight into the impact of this pandemic as well as potential future endemics, epidemics and pandemics as unfortunately, the risk of the variants of the COVID-19 virus, or a novel virus overwhelming populations remains possible.

Further, there is now cohort of families who experienced new parenting during COVID-19 and a generation of children growing up with the legacy of being born during the pandemic and the consequences this may bring. The impact of COVID-19 on the development of the babies born during the pandemic is likely to be researched widely, since as Davies-Floyd et al. [67] suggested, the pandemic provided a ‘testing ground’ for working on disaster preparedness for perinatal care both for future pandemics and other disasters.

## Electronic supplementary material

Below is the link to the electronic supplementary material.

Supplementary Material 1

Supplementary Material 2

Supplementary Material 3

## Data Availability

The data generated from this research will be stored on password protected Swansea University and NHS computers and Password protected files and devices belonging to members of the research team. Data will be kept for 10 years or up to 1 year after the findings are published in a scientific journal. All the data collected from the participants are made anonymous and held by the research institutions. The data may be made available upon request to regulatory authorities. However, the data is not shared openly as the details and circumstantial information could result in the anonymity of the participants being compromised.
