# Supplementary material for: Experiences of early parenthood in and beyond the coronavirus pandemic: a qualitative study with expectant and new mothers
Source: BMC Health Serv Res. 2025 Dec 4;26:33. doi: 10.1186/s12913-025-13746-x (PMC12781444; doi:10.1186/s12913-025-13746-x)
Supplement: Supplementary file 2 — Supplementary Material 2 [file 12913_2025_13746_MOESM2_ESM.docx]

Interview Schedule

- Would you like to tell me about circumstances of your referral to Perinatal Mental Health Services?
- Would you like to tell me about how you felt about your pregnancy/birth/postnatal period in relation to the Coronavirus outbreak?
- Was your pregnancy/birth/postnatal period during the lockdown period which started in January 2020.
- How were your experiences different because of Coronavirus?
- What did you find most useful whilst being pregnant, giving birth, being post-birth during this pandemic?
- What did you find most difficult whilst being pregnant, giving birth, being post-birth during this pandemic?
- Do you think that your mental health suffered because of being pregnant, giving birth, being within the postnatal period during this pandemic?
- How did you experience the strict lockdown measures during your pregnancy, giving birth or being within the postnatal period?
- What else would you like to tell us about your experiences?
