# Supplementary material for: Experiences of early parenthood in and beyond the coronavirus pandemic: a qualitative study with expectant and new mothers
Source: BMC Health Serv Res. 2025 Dec 4;26:33. doi: 10.1186/s12913-025-13746-x (PMC12781444; doi:10.1186/s12913-025-13746-x)
Supplement: Supplementary file 3 — Supplementary Material 3 [file 12913_2025_13746_MOESM3_ESM.docx]

## Appendix 1: Table 1: Participant Demographics

Table 1: Participant Demographics

| **Participant** | **Age of Participants** | **Occupation** | **Status During COVID-19** | **Baby’s age** | **Birth order of the baby** | **Relationship Status** |
| --- | --- | --- | --- | --- | --- | --- |
| **Swansea Bay University Health Board** | | | | | | |
| PSB01 | 30 | Business advisor | Pregnancy and post birth | 5 months | 2^nd^ | Married |
| PSB02 | 32 | Post-graduate student | Pregnancy and post birth | 10 weeks | 1^st^ | Married |
| PSB03 | 31 | Stay at home mum | Pregnancy and post birth | 9 months | 1^st^ | Married |
| PSB04 | 35 | Admin Officer | Pregnancy and post birth | 6 months | 1st | Married |
| PSB05 | 34 | Student | Post-birth | 1 year | 2^nd^ | Married |
| PSB07 | 32 | Social Worker | Pregnancy and post birth | 1 year | 1st | Co-habiting |
| PSB08 | 30 | Health care assistant | Pregnancy and post birth | 9 months | 1^st^ | Co-habiting |
| PSB09 | 34 | Receptionist | Post birth | 2 years | 2^nd^ | Married |
| PSB10 | 35 | Receptionist | Pregnancy and post birth | 6 months | 2^nd^ | Married |
| PSB11 | 32 | Ambulance services Receptionist | Post-birth | 18 months | 4^th^ | Married |
| PSB12 | 31 | Event Organiser | Pregnant | Pregnant | 1st | Co-habiting |
| PSB13 | 30 | Self-employed cake maker | Post-birth | 17 months | 1^st^ | Co-habiting |
| PSB14 | 29 | Estate Agent | Pregnancy/birth/post-birth | 11 months | 2^nd^ | Married |
| PSB15 | 23 | Support worker | Pregnancy/birth/post-birth | 12 months | 1^st^ | Co-habiting |
| PSB16 | 37 | Home mum | Post-birth | 19 months | 9^th^ &10^th^ | Co-habiting |
| **Hywel Dda University Health Board** | | | | | | |
| PHD01 | 27 | Writer | Pregnancy and post birth | 9 months | 1st | Married |
| PHD02 | 39 | Health care assistant | Pregnancy and post-birth | 4 months | 4^th^ | Married |
| PHD03 | 34 | Housewife | Pregnancy and post birth | 5 months | 2^nd^ | Married |
| PHD06 | 31 | Animal Health officer | Post birth | 18 months | 2^nd^ | Married |
| PHD07 | 38 | Education Supporting Officer | Post-covid | 15 months | 4^th^ | Married |
| PHD10 | 38 | Housewife | Pregnancy and post birth | 3 months | 2^nd^ | Married |

## Appendix 2: Table 2: Themes and Sub-themes

Table 2: Themes and Sub-Themes

| **Theme** | **Sub-themes** |
| --- | --- |
| **1. Disrupted Perinatal Medical Care during the COVID-19 Pandemic** | i. Exclusion of partners and emotional consequences  ii. Emotional distress of being alone at the hospital  iii. Home births as resistance to hospital exclusion |
| **2. Becoming a Parent in Social Isolation** | i. Unwitnessed pregnancy and the loss of rituals  ii. Fragmented social support and perinatal vulnerability |
| **3. Managing Perinatal Health Through PMHS** | i. Embodied vulnerability and anxiety due to COVID-19  ii. Mental health impact of COVID-19  iii. Remote access to PMHS during COVID-19 |
| **4. Parenthood beyond the COVID-19 Pandemic** | i. Bonding, isolation and maternal strain  ii. Different challenges for first-time and experienced mothers  iii. Fathers, bonding and mental health in pandemic |
